# Is Consumption Contagious?

**Published:** 1880-08

**Authors:** 


### IS CONSUMPTION CONTAGIOUS?

EMINENT French, English and American physicians advocate the doctrine that "consumption is catching." Morgagni, one of the greatest medical lights of his time, was such a firm believer in the opinion, that he never would assist in the examination of a person who had died of the disease. Some of the most distinguished writers as well as some of the most celebrated and successful practitioners in that disease have eventually died of it themselves, among whom were the great Laennec, Morton, Wooster, and not forgetting the empiric St. John Long, (so said.)

A large number of persons evidently consumptive will be found on inquiry to have had a husband, wife, sister, or child to have died of that disease. Statistics seem to show that a wife whose husband is consumptive, is more liable to consumptive disease than a healthy husband with a consumptive wife; the reason of this, if true, will suggest itself to the thoughtful.

Introducing the matter of small-pox into the system prevents small-pox. Laennec inoculated himself with consumptive matter but it did not "take." He subsequently died of consumption himself. He made this experiment to show that consumption was not inoculable. MM. Alberti and Biett thought that cancer was not communicable by the matter of cancer, and to prove it, tried to inoculate themselves with it but it did not "take." Both of them died afterwards from cancer.

It is most probable that consumption is not of itself communicable, that it can not beget consumption in one who has vigorous health and is perfectly free from all taint of the disease

But if any person who has not a vigorous constitution, whether inclined to consumption or not, lives, eats and sleeps with a consumptive, as man and wife do, as a sister is apt to do with a consumptive sister, or a mother with consumptive children, such persons will very generally die of consumption themselves, not from its communicability *per se*, but from the foulness of the atmosphere about a consumptive, from warm rooms, decaying lungs, large expectoration, sickening night-sweats and bodily emanations; but the same amount of exposure to air made foul in any other way would light up the fires of consumption in one of feeble vitality or broken constitution. It is best, therefore, that the nurse of a consumptive should possess the most vigorous health, and to make assurance from infection doubly sure, the most scrupulous cleanliness possible should be observed and carried out in every conceivable direction, extended to every minutiæ and maintained with the most inveterate constancy through every hour of the twenty-four, not allowing any excretion, even a single expectoration, to remain about the person, bed or room for one instant. An incessant ventilation should be going on in the chamber, the best method for which under most circumstances is simply to keep a fire on the hearth and an inner door open; even in mid-summer, this is better for the patient as well for the nurse than a room kept closed all the time from an almost insane dread of taking cold.

